# Theta-frequency subthalamic stimulation enhances conflict resolution in Parkinson’s disease patients with freezing of gait through frontal cortex modulation

**DOI:** 10.1038/s41531-025-01067-z

**Published:** 2025-07-10

**Authors:** Hutao Xie, Yutong Bai, Yutong Zhuang, Jiansong Huang, Delong Wu, Xin Zhang, Lin Shi, Hua Zhang, Jian Li, Jing Fu, Anchao Yang, Fangang Meng, Quan Zhang, Yin Jiang, Jianguo Zhang

**Affiliations:** 1 Department of Neurosurgery, Beijing Tiantan Hospital, Capital Medical University, Beijing, China; 2 Department of Functional Neurosurgery, Beijing Neurosurgical Institute, Beijing, China; 3 Department of Neurosurgery, Zhongshan Hospital (Xiamen), Fudan University, Xiamen, China

**Keywords:** Cognitive ageing, Parkinson's disease, Pathogenesis, Parkinson's disease

## Abstract

Freezing of gait (FOG) in Parkinson’s disease (PD) is a debilitating motor symptom linked to executive dysfunction, particularly impaired conflict resolution. However, the underlying neural mechanisms and optimal treatment remain unclear. We assessed conflict resolution using a modified Flanker task in 90 PD patients (52 with FOG) and 37 healthy controls. PD-FOG patients exhibited significantly greater conflict costs than patients without FOG and healthy controls. Task-based fMRI revealed enhanced frontal cortical activation associated with conflict processing deficits in PD-FOG, positively correlating with FOG severity. In a subgroup of 18 PD-FOG patients undergoing fMRI during subthalamic nucleus deep brain stimulation (STN-DBS), theta-frequency (5 Hz) stimulation improved conflict resolution and increased frontal activation, whereas high-frequency (130 Hz) stimulation primarily activated motor regions without cognitive benefit. These findings indicate that frontal dysfunction contributed to the conflict resolution deficits in PD-FOG and support theta-frequency STN-DBS as a promising therapeutic approach for enhancing cognitive function.

## Introduction

Freezing of gait (FOG) is considered one of the most debilitating symptoms that commonly occurs in advanced Parkinson’s disease (PD)^1^. FOG significantly impairs mobility, increases the risk of fall, and reduces quality of life^2,3^. Notably, FOG episodes are more frequently triggered during dual-task conditions that require concurrent cognitive-motor processing compared to simple walking tasks^4–6^. Although accumulating evidence has identified executive dysfunction as a critical contributor to FOG occurrence and shown that patients with FOG (PD-FOG) exhibit poorer executive function as compared to those without FOG (PD-nFOG)^7–10^, the precise neural mechanisms involved in FOG are not fully understood. Additionally, existing therapeutic interventions do not adequately address the cognitive impairments associated with FOG. Therefore, there is a need for improved methods to evaluate and treat these impairments to promote PD care.

Conflict resolution, a critical component of executive function, has been extensively studied in PD patients through various cognitive paradigms^7,9,11,12^. Among these, the Flanker task is one of the most widely utilized behavioral tests for assessing conflict resolution abilities^13,14^. While consistent evidence demonstrates prolonged conflict resolution times in PD patients compared to healthy controls (HCs), the findings regarding differences between PD-FOG and PD-nFOG patients remain inconclusive. Some studies have reported greater reaction time costs in PD-FOG patients compared to PD-nFOG^11,15^, whereas other investigations have failed to detect significant intergroup differences^9,16^. These inconsistent findings suggest that the conventional Flanker task (CFT) may lack sufficient sensitivity to identify conflict processing deficits specific to PD-FOG patients, underscoring the need for more refined assessment tools and targeted research approaches in this clinically relevant population.

Previous neuroimaging studies employing both structural and functional magnetic resonance imaging (MRI) have sought to elucidate the neural mechanisms underlying conflict resolution impairments in PD-FOG, consistently implicating dysfunction within the cognitive control network. Diffusion tensor imaging studies have shown structural connectivity deficits in PD-FOG patients, particularly involving disruptions affecting frontostriatal and cerebello-thalamo-cortical circuits that connect critical regions such as the prefrontal cortex (PFC), supplementary motor area (SMA), basal ganglia, thalamus, and cerebellum^5,16^. Additionally, task-based functional MRI (fMRI) studies that combined virtual walking with Stroop tasks have examined neural activity during dual-task walking in PD-FOG patients, identifying hypoactivation in key conflict-monitoring regions, including the bilateral anterior insula, ventral striatum, pre-SMA, and left subthalamic nucleus (STN), particularly when processing indirect cognitive cues during walking^17^. However, there remains a lack of evidence detailing the specific neural activity patterns during the conflict processing phase in PD-FOG patients.

Although deep brain stimulation (DBS) of STN at high frequencies (>100 Hz) is an established therapy for alleviating PD motor symptoms^18^, its clinical efficacy for FOG and related cognitive impairments is limited^19^. Notably, chronic high-frequency STN-DBS has been linked to cognitive declines in functions such as response inhibition and verbal fluency^20,21^. Given that the STN is anatomically connected to the frontal cortex via the hyperdirect pathway, and that theta-range (4–8 Hz) oscillations within this circuit are known to support cognitive processes^22^, theta frequency STN-DBS has emerged as a potential intervention to improve executive functions in patients with PD. Supporting this hypothesis, our previous study and that of others suggest that theta-range stimulation may enhance working memory^23^, verbal fluency^24^, and conflict resolution^25,26^ in PD, indicating the possibility that it could also alleviate FOG-related cognitive deficits. Nevertheless, the specific effect of theta STN-DBS on conflict resolution in PD-FOG patients has yet to be fully elucidated.

Accordingly, the present study employed a three-experiment design to systematically investigate conflict resolution deficits in PD-FOG patients, their neural correlates, and the potential modulatory effects of theta-frequency STN-DBS. Experiment 1 introduces a modified Flanker task (MFT) to more sensitively assess conflict-related impairments. Experiment 2 utilizes event-related fMRI to identify the critical brain networks involved in conflict processing and clarify how these networks differ from those in PD-nFOG patients and HCs. Finally, experiment 3 evaluates whether theta-frequency STN-DBS can improve conflict resolution and modulate the relevant neural circuits in PD-FOG patients, in comparison to traditional high-frequency stimulation. By integrating these findings, we aimed to provide novel insights into the neural mechanisms underlying FOG-related cognitive deficits and present evidence for frequency-specific STN-DBS as a targeted therapy for cognitive dysfunction in PD-FOG.

## Results

### Participant overview

In Experiment 1, 45 participants (15 PD-FOG, 15 PD-nFOG, and 15 HCs) completed behavioral testing. In Experiment 2, 64 participants (22 PD-FOG, 20 PD-nFOG, and 22 HCs) completed behavioral testing, cognitive assessments, and fMRI recording. In Experiment 3, 18 PD-FOG patients completed behavioral testing, STN-DBS, and fMRI recording (see Table 1 for details).

**Table 1:** Experimental design and participant distribution

Experiment ID	Number of participants			Experimental design
	PD-FOG	PD-nFOG	HC	
Experiment 1	15	15	15	Behavioral test
Experiment 2	22	20	22	Behavioral test, cognitive function assessment and fMRI signal recoding
Experiment 3	18	–	–	Behavioral test, STN-DBS and fMRI signal recoding

*PD-FOG* Parkinson’s disease patients with freezing of gait, *PD-nFOG* Parkinson’s disease patients without freezing of gait, *HCs* healthy controls.

### Experiment 1: Impairment of conflict resolution in PD-FOG

Demographic data (Supplementary Table S1) indicated no significant difference in age, sex, or education among the PD-FOG, PD-nFOG, and HC groups. Compared to PD-nFOG, the PD-FOG group had a longer disease duration. Cognitive assessments (MMSE, MoCA) revealed no difference between the PD-FOG and PD-nFOG groups, but both showed cognitive impairments compared to HCs.

**Fig. 1:**
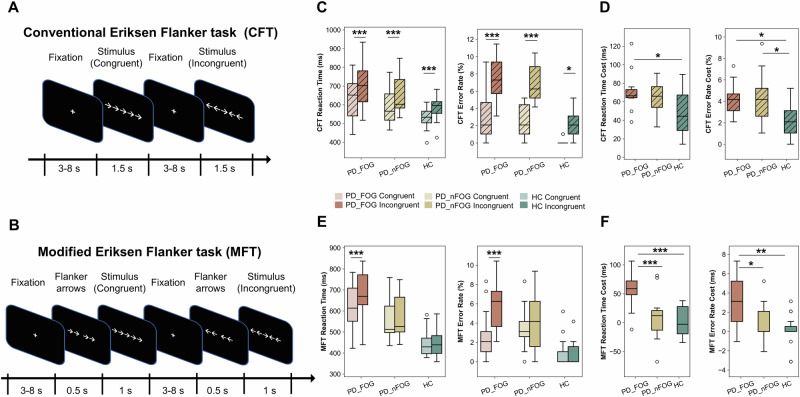
Behavioral performance in conventional and modified Eriksen Flanker tasks (Experiment 1). **A** Schematic of the Conventional Eriksen Flanker task (CFT). Participants indicated the direction of the central target arrow, flanked by congruent or incongruent arrows. **B** Behavioral results for the CFT across PD-FOG, PD-nFOG, and HC groups. All groups exhibited a significant conflict effect, with longer reaction times and higher error rates for incongruent trials compared to congruent trials. **C** One-way ANOVA of the conflict effect in the CFT. PD-FOG patients showed significantly higher reaction time and error rate costs compared to HCs, but with no significant difference compared to PD-nFOG patients. **D** Schematic of the Modified Eriksen Flanker task (MFT). Flanking arrows were presented 500 ms before the central target arrow to reduce task difficulty. **E** Behavioral results for the MFT across the three groups. A significant conflict effect was observed only in the PD-FOG group. **F** One-way ANOVA of the conflict effect in the MFT. Both reaction time and error rate costs were significantly greater in the PD-FOG group compared to the HC and PD-nFOG groups. However, no significant difference was observed between the HC and PD-nFOG groups. Bonferroni correction was applied for post-hoc comparisons. All box plots show the minimum, maximum, median, 1st quartile, and 3rd quartile. Bonferroni correction was applied for multiple comparisons in the analyses. *p*-values (asterisks) indicate the following significance levels: ^*^p < 0.05, ^**^p < 0.01, ^***^p < 0.001.

All participants sequentially completed two tasks—the CFT and MFT (Fig. 1A, B). In the CFT, all groups exhibited longer RTs and higher error rates for incongruent versus congruent trials (Fig. 1C). PD-FOG patients demonstrated significantly higher Flanker costs (RT and error rate) compared to HCs, whereas no significant difference was found between PD-FOG and PD-nFOG patients (Fig. 1D). In the MFT, increased RTs and error rates for incongruent trials were observed only in the PD-FOG group, whereas those in the PD-nFOG and HC groups showed no significant difference between trial types (Fig. 1E). Between-group analysis revealed significant differences in Flanker costs between the PD-FOG and PD-nFOG groups (Fig. 1F). Two-way repeated-measures ANOVA showed significant main effects of group and task type on RT and error rate costs, with a significant interaction for RT cost (Supplementary Fig. S1). Post hoc analyses indicated that the MFT reduced conflict costs in the PD-nFOG and HC groups but not in PD-FOG group (Supplementary Table S4).

### Experiment 2: Brain function related to conflict resolution in PD-FOG patients

Demographic, clinical, and mood measures in Experiment 2 were comparable to those in Experiment 1 (Supplementary Table S2). In cognitive assessments, both PD-FOG and PD-nFOG patients showed impairment in executive function, attention/working memory, and visuospatial ability relative to HCs, with PD-FOG patients exhibiting more pronounced deficits in executive and visuospatial domains (Table 2).

**Table 2:** Cognitive performance within the PD-FOG, PD-nFOG, and HC groups in experiment 2

	PD-FOG	PD-nFOG	HC	*p*-value	*p*-value post-hoc analysis		
Variables	(*n* = 22)	(*n* = 20)	(*n* = 22)	ANOVA	PD-FOG vs PD-nFOG	PD-FOG vs HC	PD-nFOG vs HC
Executive function							
FAB	14.64 ± 1.56	16.05 ± 1.19	16.82 ± 0.85	<0.001*	<0.001*	<0.001*	0.147
Stroop color (seconds)^c^	47.00 ± 13.35	42.60 ± 10.30	34.50 ± 6.54	<0.001*	0.831	<0.001*	0.018*
Stroop interference (seconds)	95.18 ± 25.20	80.15 ± 10.12	61.68 ± 11.21	<0.001*	0.018*	<0.001*	0.003*
TMT B (seconds)	216.77 ± 84.50	192.15 ± 41.33	127.77 ± 26.84	<0.001*	0.499	<0.001*	0.002*
VFT-nonepisodic	16.05 ± 3.42	16.90 ± 5.43	22.14 ± 4.45	<0.001*	>0.999	<0.001*	0.001*
VFT-episodic	14.73 ± 3.87	15.80 ± 4.80	21.36 ± 4.54	<0.001*	>0.999	<0.001*	<0.001*
VFT-switch	13.09 ± 2.71	13.70 ± 4.65	17.77 ± 3.44	0.001*	>0.999	<0.001*	0.002*
Attention and working memory							
TMT B-A (seconds)^c^	115.73 ± 62.91	98.70 ± 24.35	65.68 ± 20.84	0.001*	>0.999	0.008*	<0.001*
TMT A (seconds)	101.05 ± 31.30	93.45 ± 22.89	62.09 ± 18.64	<0.001*	0.982	<0.001*	<0.001*
SDMT^c^	24.14 ± 9.05	26.75 ± 6.77	36.41 ± 8.58	<0.001*	>0.999	<0.001*	0.005*
DST-backward^c^	3.05 ± 1.01	3.20 ± 1.02	3.96 ± 1.05	0.015*	>0.999	0.021*	0.069
Visuospatial							
JLO	18.45 ± 2.81	21.50 ± 2.54	24.18 ± 2.68	<0.001*	0.032*	<0.001*	0.026*
TPCP^c^	7.82 ± 1.33	8.95 ± 1.10	9.73 ± 0.70	<0.001*	0.030*	<0.001*	0.043*
Language							
Boston Naming Test^c^	25.82 ± 2.13	26.05 ± 2.46	27.41 ± 2.04	0.044	>0.999	0.060	0.153
Memory							
DST-forward^c^	5.30 ± 1.05	5.40 ± 1.21	5.68 ± 0.72	0.367	>0.999	0.556	0.836
AVLT-20 min delayed recall^c^	4.32 ± 1.29	5.50 ± 2.65	5.84 ± 1.73	0.019*	0.442	0.014*	0.573
AVLT-Recognition^c^	21.05 ± 1.76	21.25 ± 2.53	22.59 ± 1.14	0.011*	>0.999	0.009*	0.150

Values are presented with means ± standard deviations; p-value refers to one-way ANOVA followed by post hoc Bonferroni’s multiple comparisons tests or to ^c^Kruskall–Wallis test followed by post hoc Dunn test. *Groups significantly different at p < 0.05.

*PD-FOG* Parkinson’s disease patients with freezing of gait, *PD*-n*FOG* Parkinson’s disease patients without freezing of gait, *HC*s healthy controls, *FAB* Frontal Assessment Battery, *VFT* Verbal Fluency Test, *TMT B-A* Time to Complete the Trail Making Test Part B minus Part A, *SDMT* Symbol Digit Modalities Test, *DST*-backward Verbal Digital Span Test-Backward, *JLO* Benton’s Judgement of Line Orientation, *TPCT* Ten-Point Clock Test, *DST*-forward verbal Digital Span Test-forward, *AVLT* Auditory Verbal Learning Test.

**Fig. 2:**
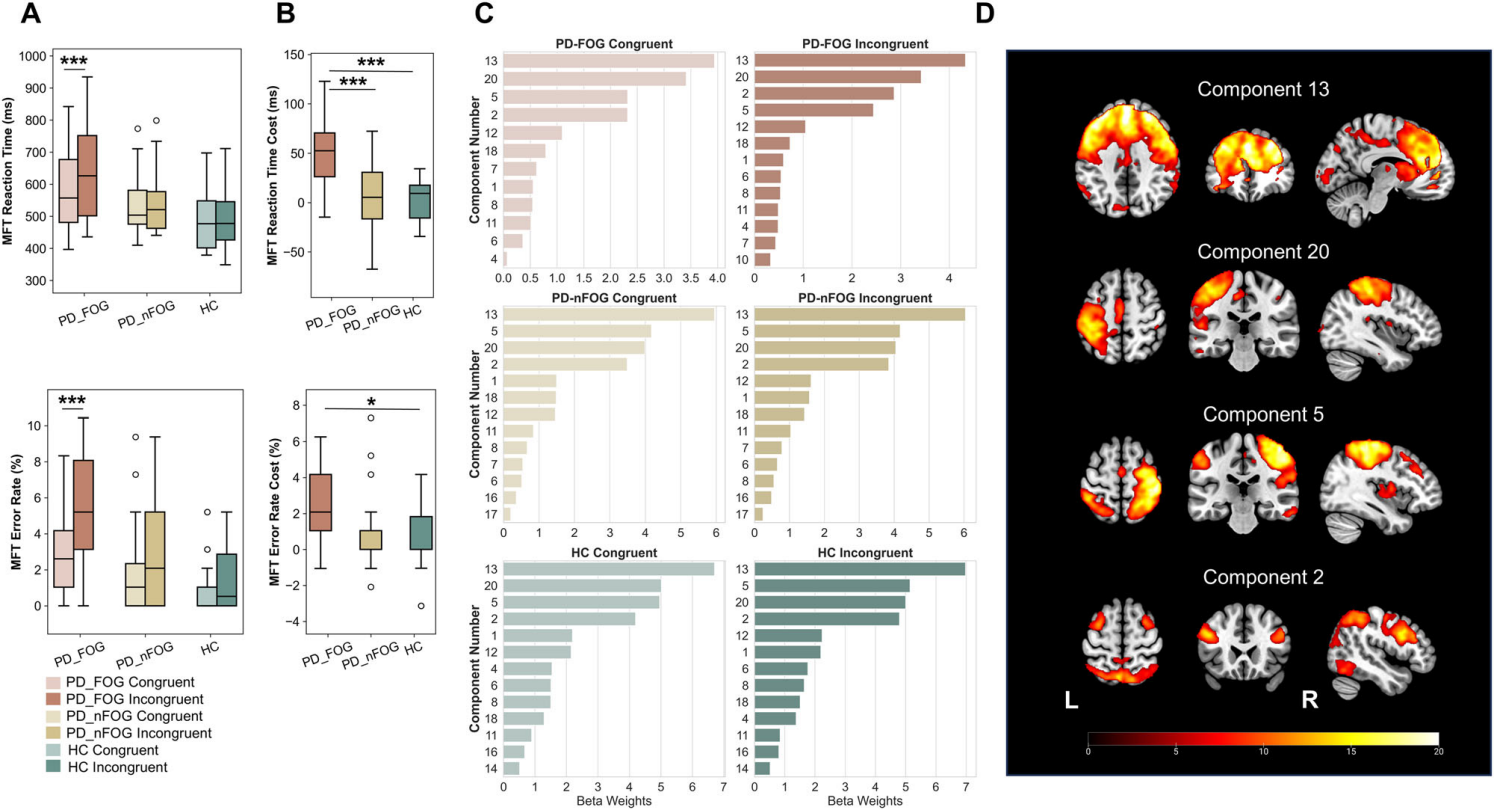
Brain networks related to cognitive conflict resolution in PD-FOG patients (Experiment 2). **A** Behavioral results of the MFT for the PD-FOG, PD-nFOG, and HC groups. Similar to Experiment 1, a significant conflict effect was observed only in the PD-FOG group, with longer reaction times and higher error rates for incongruent trials compared to congruent trials. **B** One-way ANOVA of the conflict effects across the three groups. The PD-FOG group exhibited significantly greater reaction time and error rate costs compared to the HC and PD-nFOG groups. However, no significant difference was observed between the HC and PD-nFOG groups. **C** Independent component analysis (ICA) of task-based fMRI data identified 20 components. Components 13, 20, 5, and 2 were most strongly associated with the MFT. **D** Spatial distribution of brain regions involved in components 13, 20, 5, and 2. Component 13 primarily included the bilateral dorsolateral prefrontal cortex (PFC), pre-SMA, and dACC; component 20 included the left motor cortex (M1); component 5 encompassed the right M1; and component 2 involved bilateral prefrontal and parietal cortices. All box plots show the minimum, maximum, median, 1st quartile, and 3rd quartile. Bonferroni correction was applied for multiple comparisons in the analyses. *p*-values (asterisks) indicate the following significance levels: ^*^p < 0.05, ^***^p < 0.001.

During fMRI scanning while performing the MFT, behavioral performance replicated Experiment 1. Significant differences in RTs and error rates between incongruent and congruent trials were observed only in the PD-FOG group, resulting in higher Flanker costs compared to the other groups (Fig. 2A, B, Supplementary Table S5).

ICA identified four task-related components (components 13, 20, 5, and 2) (Fig. 2C). These components mainly included the dorsolateral PFC (MFG and IFG), medial PFC [medial superior frontal gyrus (SFG) extending to the dACC and pre-SMA], primary motor cortex (M1), and parietal lobe. Specifically, component 13 primarily included the bilateral dorsolateral PFC, pre-SMA, and dACC. In addition, component 20 primarily included the left M1, component 5 encompassed the right M1, and component 2 involved the bilateral prefrontal and parietal cortices (Fig. 2D).

**Fig. 3:**
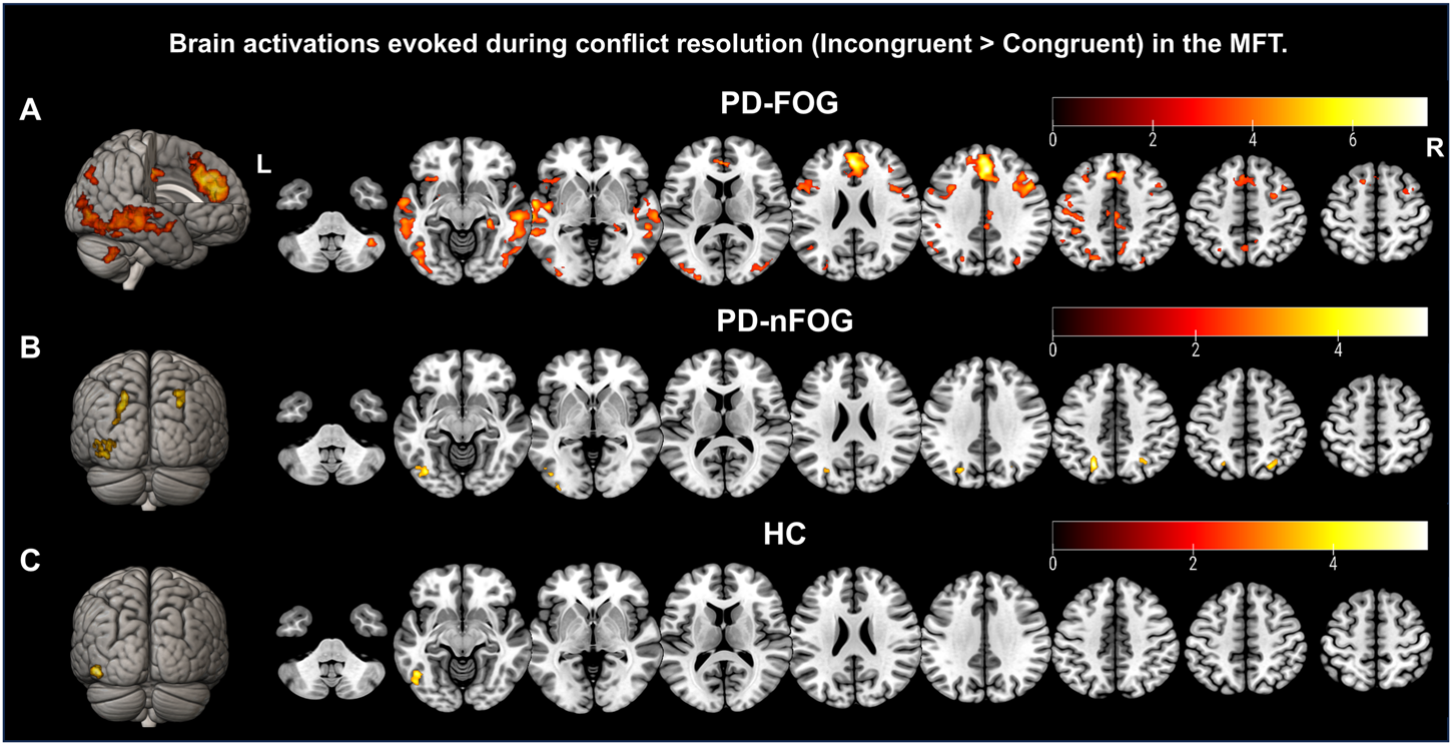
Whole-brain activation patterns during the conflict effect (incongruent > congruent) for the MFT (Experiment 2). **A** PD-FOG group: significant activation was observed in regions including the pre-SMA, dACC, medial SFG, and bilateral MTG. **B** PD-nFOG group: Activation primarily observed in the occipital cortex and superior parietal lobe (SPL). **C** HC group: Activation in the inferior temporal gyrus, occipital cortex, and fusiform gyrus. Group-level comparison of activation differences. The PD-FOG group exhibited significantly higher activation in the dACC, bilateral MTG, right MFG, and posterior cingulate cortex (PCC) compared to the PD-nFOG and HC groups.

**Fig. 4:**
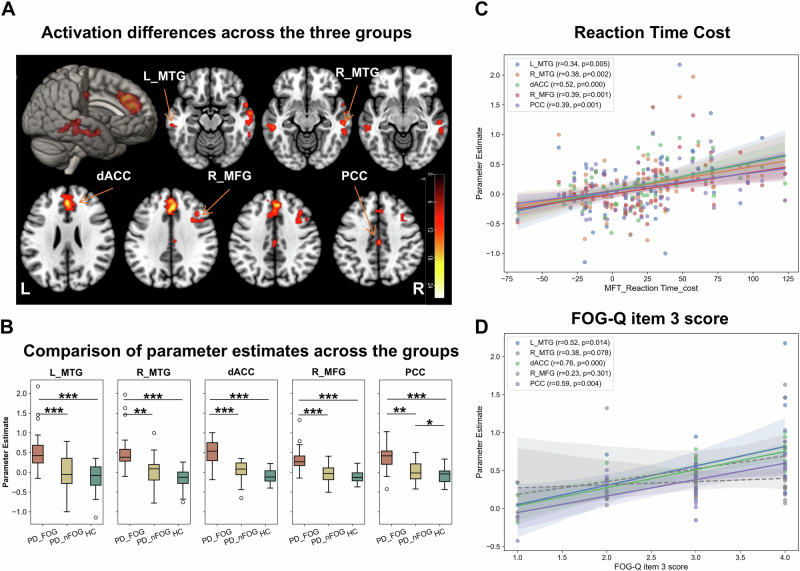
Group-level analysis of activation differences and correlations with behavioral measures (Experiment 2). **A** Activation differences across the three groups, with significant clusters in the dACC, bilateral MTG, right MFG, and PCC. **B** Parameter estimates extracted from the identified brain regions. The PD-FOG group showed significantly higher activation levels compared to the PD-nFOG and HC groups in all regions. **C** Brain-behavior correlation analysis: Spearman’s correlation analysis across all participants showed significant correlations between activation in the identified regions (dACC, MTG, MFG, and PCC) and reaction time costs. **D** Correlation analysis within the PD-FOG group: Significant correlations between activation intensity in dACC, left MTG, and PCC with severity of FOG (FOG-Q item 3 score). All box plots show the minimum, maximum, median, 1st quartile, and 3rd quartile. *p*-values (asterisks) indicate the following significance levels: ^*^p < 0.05, ^**^p < 0.01, ^***^p < 0.001.

Whole-brain analysis of the contrast “incongruent > congruent” showed that PD-FOG patients had significant activation in the pre-SMA, dACC, medial SFG, and bilateral middle temporal gyrus (MTG) (Fig. 3A). In contrast, PD-nFOG patients and HCs exhibited activation primarily in the occipital cortex and superior parietal lobe (SPL; Fig. 3B, C, Supplementary Table S6). Group-level comparisons revealed significantly higher activation in the dACC, right MFG, bilateral MTG, and posterior cingulate cortex (PCC) in PD-FOG patients compared to the other groups (Fig. 4A, B, Supplementary Table S6). Moreover, parameter estimates from these regions correlated positively with RT cost across subjects (Fig. 4C), and within the PD-FOG group, activation in the dACC, left MTG, and PCC correlated with FOG severity (Fig. 4D).

### Experiment 3: modulation of conflict resolution by theta frequency STN-DBS

**Fig. 5:**
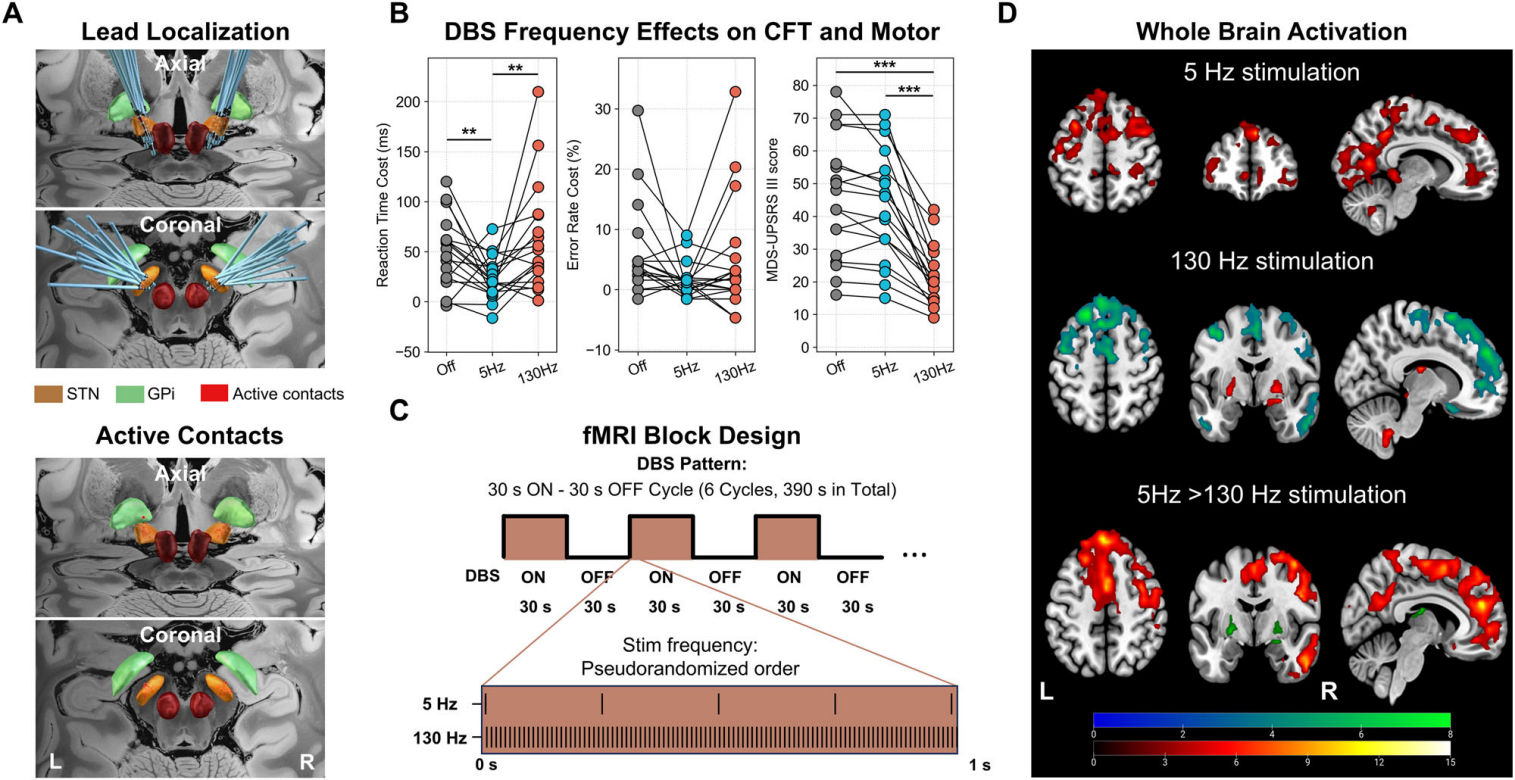
Effects of different frequency DBS-STN stimulation on behavioral and motor response and BOLD signal (Experiment 3). **A** DBS electrode localizations in 18 PD-FOG patients (top), reconstructed using Lead-DBS v3.0. Active contacts (bottom) are shown in MNI space as point-cloud representations and overlaid on an ultrahigh-resolution human brain template. Electrode positions were referenced to the DISTAL atlas. **B** Behavioral and motor performance on the conflict task and motor symptom scores across stimulation conditions. Theta-frequency (5 Hz) stimulation significantly reduced the conflict effect (reaction time cost) compared to the DBS OFF condition, whereas high gamma (130 Hz) stimulation did not significantly affect conflict processing. In contrast, high gamma stimulation significantly improved motor symptoms relative to the DBS OFF condition, while theta-frequency stimulation did not produce a significant motor benefit. See Supplementary Table S7 for details. **C** The fMRI block design: DBS ON/OFF cycles (30 s ON, 30 s OFF) synchronized with fMRI acquisition. Stimulation frequencies (5 Hz and 130 Hz) were applied in a pseudorandomized, counterbalanced order within subjects. **D** Whole-brain BOLD response maps during DBS stimulation. Theta stimulation activated frontal, parietal regions, and deep cerebellar nuclei, including the bilateral medial SFG, bilateral MFG, left IFG, orbitofrontal cortex, bilateral angular gyrus, and cerebellar posterior lobe. High gamma stimulation activated the thalamus, GPi, and cerebellum, inhibiting the medial SFG, pre-SMA/dACC, bilateral IFG, and MTG. Color bar shows T-value range. Detailed cluster information is provided in Supplementary Table S6. Number of voxels exceeding a voxel-level threshold of *p* < 0.001, with cluster-level *p* < 0.05, FWE-corrected for multiple comparisons.

All 18 PD-FOG patients in Experiment 3 met the criteria for the postural instability and gait difficulty (PIGD) subtype followed by established criteria^27^. Electrode reconstruction showed that all active DBS contacts were located within or immediately adjacent to the STN (Fig. 5A). Theta-band (5 Hz) stimulation significantly reduced the Flanker cost compared to the DBS OFF condition, while high gamma (130 Hz) stimulation had no behavioral effect. In contrast, only 130 Hz stimulation significantly improved MDS-UPDRS III motor scores, with no significant motor benefit observed at 5 Hz (Fig. 5B, Supplementary Fig. S2 and Supplementary Table S7). The fMRI analysis under theta stimulation (DBS ON > OFF) showed increased activation in frontal and parietal regions and deep cerebellar nuclei, including the bilateral medial SFG, bilateral MFG, left triangular IFG, orbitofrontal cortex, bilateral angular gyrus, and cerebellar posterior lobe (Fig. 5C, D, Supplementary Table S8). In contrast, high gamma stimulation resulted in increased activation in the bilateral thalamus, globus pallidus internus (GPi), and deep cerebellar nuclei, along with decreased activation in frontal and temporal regions (including medial SFG extending to pre-SMA/dACC, bilateral IFG, and MTG). Direct comparisons indicated that theta stimulation preferentially activated frontal and parietal regions, whereas high gamma stimulation predominantly engaged subcortical motor structures (Fig. 5D).

## Discussion

In the present study, we employed an MFT paradigm to elucidate conflict processing mechanisms in PD-FOG patients and identified several key findings. First, such patients demonstrated significant impairments in conflict processing, as assessed by the MFT compared to both PD-nFOG patients and HCs. Second, in PD-FOG patients, activation levels in the frontal cortex were associated with conflict processing and exhibited a positive correlation with conflict effects. Notably, the activation level in the cingulate cortex also showed a positive correlation with the severity of FOG. Third, theta-frequency STN-DBS enhanced conflict resolution abilities in PD-FOG patients, as evidenced by modulated activity of frontal cortex.

Executive-attentional dysfunction in PD-FOG patients has been widely reported, yet most previous studies have relied primarily on cognitive assessment scales, with few utilizing paradigms specifically designed to address FOG-related cognitive deficits^7–9^. The CFT is a commonly used measure of conflict processing that assesses attention and response inhibition^13,14^. However, studies examining Flanker performance in PD-FOG patients have yielded mixed findings. For instance, Vandenbossche et al. reported an increased Flanker effect (i.e., greater reaction time cost) in PD-FOG patients compared to PD-nFOG patients and HCs^11,15^. In contrast, other studies, including those by Cohen et al.^9^, Fling et al.^16^, and the present study, found no significant difference in the Flanker effect between PD-FOG and PD-nFOG patients. These inconsistent results collectively suggest that the CFT may lack the sensitivity required to detect conflict processing deficits specifically associated with PD-FOG. To address this limitation, we implemented the MFT, which included a 500 ms delay for the target arrow to reduce conflict salience^14^. This modification effectively eliminated the conflict effect in both HCs and PD-nFOG patients, as indicated by similar reaction times and error rates for incongruent and congruent trials. In contrast, PD-FOG patients continued to exhibit a pronounced conflict effect, demonstrating that the MFT may be more sensitive in detecting conflict processing deficits in FOG patients. Moreover, the consistency of the MFT results obtained during MRI scanning and seated testing underscores its potential as a reliable tool for future research into conflict resolution impairments in PD-FOG patients.

Our findings provided additional evidence supporting the role of the fronto-parietal network in cognitive processing tasks among PD-FOG patients. This aligned with previous studies highlighting the involvement of regions like the dACC and pre-SMA in conflict resolution during motor tasks, further emphasizing the role of cognitive control in FOG episodes^11,17^. We found that PD-FOG patients demonstrated widespread activation across a fronto-parietal-occipital network during the MFT. In agreement with prior studies, key regions involved included the dACC, pre-SMA, DLPFC, and IPL^28–32^. These regions are critical for conflict monitoring^33^ and top-down attention control^34^, suggesting that PD-FOG patients require additional cognitive resources to manage conflict during motor tasks. In contrast, PD-nFOG patients and HCs primarily showed activation of the parieto-occipital cortex, relying predominantly on attention networks without significant engagement of conflict resolution mechanisms^35^. Group difference analyses revealed that PD-FOG patients showed significantly greater activation in the medial PFC, DLPFC, and PCC, with activation levels positively correlating with the severity of FOG. These findings suggested that PD-FOG patients engaged in both conflict detection and resolution during the MFT, whereas PD-nFOG patients and HCs processed stimuli primarily through attentional network.

In Experiment 3, we further evaluated the impact of theta-frequency STN-DBS on conflict resolution in PD-FOG patients, using the CFT instead of the MFT. The CFT is a classic response conflict paradigm that provides a clearer conflict effect, thereby enhancing the visibility of therapeutic effects and minimizing ceiling effects^14^. To ensure consistency across the experiment, we maintained a constant stimulation amplitude to achieve stable volumes of tissue activated^23^. Our comparison of theta (5 Hz) and high-gamma (130 Hz) stimulation revealed that theta stimulation significantly reduced the conflict effect (reaction time cost) in PD-FOG patients performing the CFT. These findings are consistent with prior studies showing that theta-frequency STN stimulation improves conflict resolution in PD^25^. Furthermore, recent research suggests that theta-frequency stimulation can also improve other executive functions, such as working memory^23^ and verbal fluency^24^, indicating that theta stimulation has the potential to enhance multiple subsets of executive function in PD-FOG patients.

Previous studies have shown that low-frequency oscillations (4–8 Hz) within the medial PFC-STN hyperdirect circuit play a critical role in cognitive control, and that theta-frequency STN-DBS enhances executive performance by increasing theta power in the PFC in PD patients^22,36–39^. Consistent with this, our fMRI results revealed that theta stimulation significantly activated prefrontal regions, including the medial SFG, extending to the pre-SMA/dACC, bilateral dorsolateral SFG, and right IFG. In contrast, high-gamma stimulation activated primarily subcortical motor circuits (bilateral thalamus and GPi), aligning with prior findings linking high-frequency STN-DBS to motor improvements via the GPi-thalamus-cerebellum pathway^40^.

Clinically, these frequency-specific effects support the potential of a dual-frequency DBS strategy that applies high gamma stimulation to improve motor function and theta-frequency stimulation to enhance cognitive control. Given the functional segregation of the subthalamic nucleus, with dorsal regions associated with motor processing and ventral regions with cognitive functions^41^, future studies should investigate simultaneous gamma stimulation in the dorsal STN and theta stimulation in the ventral STN. Furthermore, advances in adaptive DBS algorithms that enable real-time parameter adjustments based on cognitive and motor states offer promising opportunities for personalized treatment in PD^42^.

Our study had several limitations. First, the fMRI task design in Experiment 3 utilized a block design rather than an event-related design due to the low temporal resolution of the 1.5 T MRI scanner, a choice necessitated by the MRI compatibility constraints of the DBS equipment. As 3 T MRI-compatible DBS devices become more readily available, future studies should consider to validate these findings with higher-resolution MRI. Second, the assessment of FOG severity in PD patients was based on the FOG-Q, a patient-reported measure, rather than objective quantitative metrics. Future research can improve the accuracy of FOG assessments by incorporating advanced gait analysis tools for a more precise and quantitative evaluation of FOG severity. Third, only the PIGD subtype of PD-FOG patients were included in the fMRI experiments. This criterion was adopted to reduce motion artifacts to ensure data quality, and to reduce clinical heterogeneity of the patient cohort, but it may limit the generalizability of our findings to tremor-dominant phenotypes. Future studies should aim to include a broader range of motor subtypes to comprehensively evaluate the frequency-dependent effects of STN-DBS. Fourth, Experiment 1 and 2 were conducted in the medication-ON state to improve patient compliance and reduce motion-related artifacts, whereas Experiment 3 was conducted in the medication-OFF state to parse out DBS-induced functional brain changes without the confounding effect of medication. Although PD-FOG patients exhibited conflict processing deficits linked to frontal dysfunction, and theta-frequency STN-DBS improved both behavioral performance and frontal activation, the potential influence of dopaminergic medication cannot be fully excluded. The cognitive impact of dopaminergic therapy in PD is complex and remains incompletely understood. Notably, excessive dopamine may impair flexible cognitive control due to an inverted U-shaped relationship between dopamine levels and prefrontal cortex function^43–45^. Future studies with medication-balanced or within-subject crossover designs are warranted to further clarify this interaction.

In conclusion, this study identified significant deficits in conflict resolution in PD-FOG patients, while also establishing a robust behavioral paradigm for future investigations. The neuroimaging findings underscored the pivotal role of the frontal cortex in conflict processing deficits observed in PD-FOG patients. Additionally, theta-frequency STN-DBS appeared to mitigate these deficits by modulating frontal cortex activity, highlighting the potential of STN-DBS as a promising therapeutic approach for enhancing cognitive function in PD-FOG patients.

## Methods

### Study design and participants

This study comprised three experiments designed to investigate conflict resolution deficits in PD-FOG patients, their neural correlates, and the frequency effects of STN-DBS. Experiment 1 developed and validated a modified Flanker task to detect cognitive conflict impairments sensitively in PD-FOG patients. Experiment 2 combined the MFT with event-related fMRI to identify brain regions associated with impaired conflict processing in PD-FOG. Experiment 3 evaluated the behavioral and neural impact of STN-DBS at different stimulation frequencies (5 Hz and 130 Hz) on conflict resolution in 18 PD-FOG patients, utilizing a within-subject design and fMRI under each stimulation condition.

A total of 90 PD patients and 37 HCs, aged 50–80 years, participated in this study, which comprised three experiments. All PD patients were evaluated by a movement disorders specialist and fulfilled the following criteria: (1) diagnosis of idiopathic PD according to the MDS clinical diagnostic criteria^46^, and (2) absence of other neurological disorders. Based on the definition from previous studies^47,48^, patients were determined to have PD-FOG if they (1) had a score of ≥1 on item 3 of the Freezing of Gait Questionnaire (FOG-Q) and (2) exhibited at least one freezing episode during clinical assessments (e.g., Timed Up & Go test). Patients with severe tremors were excluded from Experiments 2 and 3 to ensure the quality of the MRI data. Detailed participant numbers for each experiment are presented in Table 1, and demographic information is provided in Supplementary Tables S1–S3. The study was approved by the Ethics Committee of Beijing Tiantan Hospital (approval number: KY 2018-008-01) and conducted in accordance with the principles of the Declaration of Helsinki. All participants provided written informed consent prior to participation.

### Clinical and neuropsychological assessments

Two trained neurologists administered all clinical and neuropsychological assessments. PD motor severity was evaluated using the Hoehn and Yahr scale and Movement Disorder Society Unified Parkinson’s Disease Rating Scale part III, while FOG severity was assessed with the FOG-Q. Additional clinical data, including levodopa equivalent daily dose, disease duration, and side of onset, were recorded^49^.

To minimize the influence of motor symptoms on cognitive performance, neuropsychological testing was performed during the medication “on” state. Global cognition was assessed using the Mini-Mental State Examination (MMSE) and Montreal Cognitive Assessment (MoCA). Anxiety and depression were measured using the 14-item Hamilton Anxiety Rating Scale and the 24-item Hamilton Depression Rating Scale, respectively. Cognitive function was further evaluated across five domains: (1) executive function, which was assessed using the Frontal Assessment Battery, Stroop Color-Word Test, and Verbal Fluency Test; (2) attention and working memory, which was assessed using the Digit Span Test-Backward, Trail Making Test (Part B minus Part A), and Symbol Digit Modalities Test; (3) visuospatial function, which was assessed using the Benton’s Judgement of Line Orientation, Ten-Point Clock Test; (4) memory, which was assessed using the Digit Span Test-Forward, and Auditory Verbal Learning Test; and (5) language, which was assessed using the Boston Naming Test.

### Experiment 1: task design

The CFT was administered on a laptop using E-Prime 3.0 (Psychology Software Tools, Pittsburgh, PA, USA). Each trial began with a 500 ms fixation cross (“+”, 0.85° in diameter), followed by five arrows. In congruent trials, all arrows pointed in the same direction (→→→→→ or ←←←←←), whereas in incongruent trials, the central arrow pointed opposite to the flanking arrows (e.g., →→←→→ or ←←→←←) (Fig. 1A). Participants responded as quickly and accurately as possible using an Xbox controller to indicate the direction of the central arrow. To enhance sensitivity to conflict processing deficits, we developed an MFT in which the flanking arrows were presented 500 ms before the central target arrow, which then appeared for 1000 ms^14,50^ (Fig. 1B). This modification was designed to reduce the conflict effect in HCs and thereby unmask deficits in PD-FOG patients.

Both the CFT and MFT were structured into three runs, each lasting 6 min and 51 s. Each run began with a 10,500 ms fixation period, followed by 64 pseudorandomly presented trials equally divided into four target types (Congruent Left, Congruent Right, Incongruent Left, and Incongruent Right) with interstimulus intervals ranging from 3000 to 8000 ms (mean = 4500 ms)^29^. Participants were instructed to respond within 2000 ms; trials without a response were marked as missed. Prior to the main task, subjects completed at least 36 practice trials (18 congruent, 18 incongruent).

### Experiment 1: task performance

Task performance was quantified by mean reaction time (RT; excluding error trials and RTs <200 ms), error rate (%), RT cost (incongruent minus congruent), and error rate cost (incongruent minus congruent), with the latter two indices representing the conflict effect^28,51–53^.

### Experiment 2: MRI data acquisition

Structural and fMRI data were acquired on a 3T Siemens Prisma scanner (Siemens Healthcare, Erlangen, Germany) using a 64-channel head coil. All PD patients were scanned in the medication “on” state. In a single session, participants underwent one structural scan and three functional runs (each 6 min 50 s) while performing the MFT. Visual stimuli were presented via an LCD projector (SA-9900 fMRI Stimulation System, manufactured by Shenzhen Sinorad Medical Electronics, Inc., Shenzhen, Guangdong, China) and viewed through a mirror attached to the head coil.

Structural images were obtained using a 3D T1-weighted MPRAGE sequence (176 sagittal slices; TR = 1560 ms; TE = 1.69 ms; flip angle = 8°; matrix = 256 × 256; slice thickness = 1 mm; voxel size = 1 × 0.94 × 0.94 mm). BOLD images were acquired using an echo-planar imaging (EPI) sequence (TR = 1500 ms; TE = 30 ms; flip angle = 70°; FOV = 220 × 220 mm²; matrix = 64 × 64; multiband factor = 4; 72 slices; voxel size = 2 × 2 × 2 mm; 263 volumes per run), preceded by 11 dummy scans.

### Experiment 2: the fMRI data preprocessing

Data were preprocessed using SPM12 (http://www.fil.ion.ucl.ac.uk/spm) and MATLAB (MathWorks, Natick, MA, USA). Preprocessing steps included visual inspection for artifacts, slice-timing correction, realignment for head motion correction, co-registration to the 3D T1 image, normalization to Montreal Neurological Institute (MNI) space (2 mm isotropic voxels), and spatial smoothing with a 6 mm FWHM Gaussian kernel. Subjects with head motion exceeding 2 mm translation or 2° rotation in any run were excluded.

### Experiment 2: MFT-related network analysis

A general linear model (GLM) was constructed in SPM12 including six regressors per run (baseline, congruent, incongruent, congruent errors, incongruent errors, and missed responses), convolved with the can onical hemodynamic response function; six motion parameters were also included. Group-level Independent Component Analysis (ICA) was performed using the Group ICA of fMRI Toolbox (GIFT V4.0; http://icatb.sourceforge.net/), with the number of components set to 20 based on minimum description length criteria. Back-reconstruction yielded individual z-maps, which were compared using one-sample t-tests. Temporal profiles of ICA components were then correlated with the task design via multiple regression analyses to identify components most relevant to the MFT.

### Experiment 2: conflict-related activation analysis

For each subject, the contrast “incongruent > congruent” was computed to isolate conflict processing^54,55^. Second-level analyses (one-sample *t*-tests per group followed by one-way ANOVA across groups) identified brain regions exhibiting differential activation. Regions of interest (ROIs) were defined as 8 mm spheres centered on peak activation coordinates using the Marsbar toolbox (http://marsbar.sourceforge.net/). Spearman rank correlations were performed between behavioral conflict effects (RT cost) and ROI activation, and within the PD-FOG group, correlations between activation and FOG-Q item 3 scores were examined.

### Experiment 3: DBS leads localization and stimulation settings

Eighteen PD-FOG patients with implanted STN-DBS systems (model 3389, Medtronic) were enrolled. Electrode localization was performed using the Lead-DBS v3.0^56^ (http://www.lead-dbs.org) following established procedures^57^. Briefly, postoperative CT scans were linearly co-registered to preoperative T1-weighted MRIs using Advanced Normalization Tools (http://stnava.github.io/ANTs/) and normalized to MNI space using the SyN algorithm^58^. Brain shift correction was applied due to possible pneumocephalus. Electrode trajectories were automatically reconstructed using the PaCER^59^ or TRAC/CORE^60^ algorithms and manually refined when necessary. Active contacts were localized relative to the STN as defined by the DISTAL atlas^61^. To improve spatial clarity, point-cloud representations of active contacts were also generated.

DBS settings and dopaminergic medication regimens were optimized and stabilized by an independent neurologist. To minimize dopaminergic medication effects on cognitive control^62^, the computerized CFT was conducted in the medication “off” state. The task was performed under three stimulation conditions: DBS off, theta-frequency (5 Hz), and high gamma-frequency (130 Hz). The condition order was pseudorandomized and counterbalanced across participants to minimize order and carryover effects. All stimulation parameters, except frequency, were held constant to ensure comparable volumes of tissue activated. Active contacts were primarily selected based on clinical efficacy during postoperative programming. The Lead-DBS toolbox was used to localize the DBS leads and active contacts relative to the STN. After completing 25-min cognitive sessions (with ≥1-h wash-in/rest between conditions), patients underwent fMRI scanning using a 30 s DBS ON/30 s DBS OFF cycling paradigm.

### Experiment 3: MRI data acquisition

MRI scans were performed on a 1.5T GE Signa Explorer scanner (General Electric, San Ramon, CA, USA). Structural images were acquired using an MPRAGE sequence (286 sagittal slices; TR = 1146 ms; TE = 4.97 ms; flip angle = 12°; matrix = 256 × 256; slice thickness = 1 mm; voxel size = 1 × 1 × 0.7 mm). Functional images were obtained using an EPI sequence (TR = 2500 ms; TE = 35 ms; flip angle = 90°; FOV = 240 × 240 mm²; matrix = 64 × 64; 42 slices; voxel size = 3.75 × 3.75 × 4 mm; 156 volumes per run).

The experimental demands in Experiment 3 differed substantially from those in Experiment 2. Unlike the single scanning session in Experiment 2, each participant in Experiment 3 underwent two separate fMRI sessions under different DBS frequencies (5 Hz and 130 Hz), with at least 60 min between sessions to prevent frequency carryover effects. Furthermore, all scans in Experiment 3 were performed in the medication-OFF state to minimize pharmacological confounds. These factors prolonged the scanning schedule and increased patient burden, requiring stricter selection criteria. For safety and compatibility, Experiment 2 was conducted on a 3T scanner in patients without implanted devices, while Experiment 3 was conducted on a 1.5T system in post-DBS patients (model 3389, Medtronic).

Each stimulation condition (5 Hz, 130 Hz) included one structural scan and two functional runs (6 min 30 s each), for a total of four functional runs per participant. Each fMRI run employed a block design composed of alternating 30-second DBS ON and OFF epochs, starting with an ON block. DBS cycling (30 s ON/30 s OFF) was embedded within each individual frequency condition, such that the ON and OFF blocks were confined to the same stimulation frequency. The order of stimulation conditions was pseudorandomized and counterbalanced across participants to mitigate order and carryover effects. The DBS ON/OFF cycling was manually synchronized with fMRI acquisition, following established protocols^63,64^.

Notably, the OFF blocks served as within-condition baselines for their respective stimulation frequencies. For each frequency, the ON > OFF contrast was computed independently, using only the ON and OFF blocks from that frequency’s own runs. No OFF periods were pooled or compared across frequencies, in order to avoid potential confounds such as temporal drift, frequency-specific baseline shifts, or residual carryover effects between stimulation conditions.

### Experiment 3: DBS-induced activation estimation

Due to magnetic susceptibility artifacts primarily affecting the left hemisphere (inferior parietal, temporal, occipital lobes, and cerebellum), enantiomorphic normalization^65,66^ was applied prior to standard preprocessing (removal of the first 12 volumes, slice timing correction, motion correction, rigid registration to T1-weighted images, normalization to MNI space, and smoothing with an 8 mm FWHM Gaussian kernel in SPM12).

For each stimulation frequency, we constructed a separate general linear model (GLM) comprising two block regressors (30-second DBS ON and OFF periods) and six motion parameters as nuisance covariates. A high-pass filter with a cutoff of 128 s was applied to remove low-frequency drift. The primary contrast of interest (ON > OFF) was estimated independently for each frequency condition, using only the ON and OFF epochs within that specific condition. Contrast images from each participant were entered into group-level one-sample t-tests to identify consistent DBS-induced activation patterns.

### Statistical analysis

Continuous variables are reported as the mean ± standard error. Statistical analyses and data visualizations were performed in Python 3.12 (Python Software Foundation, https://www.python.org). For Experiments 1 and 2, group demographic differences were assessed using one-way ANOVA or chi-squared tests, while clinical variables were compared using independent *t*-tests or Mann–Whitney U tests as appropriate. Task performance metrics (RT and error rate) were analyzed with a 2 (congruency) × 3 (group) mixed-model ANOVA. Differences in Flanker cost and fMRI activation were evaluated with one-way ANOVA. In Experiment 3, repeated-measures ANOVA was used to assess Flanker cost differences across stimulation frequencies and paired t-tests to compare whole-brain activation between frequencies. Bonferroni corrections were applied for multiple comparisons in both behavioral and fMRI analyses. Voxel-level threshold of *p* < 0.001 with a cluster-level FWE-corrected threshold of *p* < 0.05 was used for all fMRI analyses.

## Supplementary information

Supplementary Information

## Data Availability

The datasets generated during and/or analyzed during the current study are available from the corresponding author on reasonable request.
