# Supplementary material for: Theta-frequency subthalamic stimulation enhances conflict resolution in Parkinson’s disease patients with freezing of gait through frontal cortex modulation
Source: NPJ Parkinsons Dis. 2025 Jul 10;11:206. doi: 10.1038/s41531-025-01067-z (PMC12246457; doi:10.1038/s41531-025-01067-z)
Supplement: Supplementary file 1 — Supplementary Information [file 41531_2025_1067_MOESM1_ESM.docx]

**Supplementary Materials**

**Supplementary Figures**

**
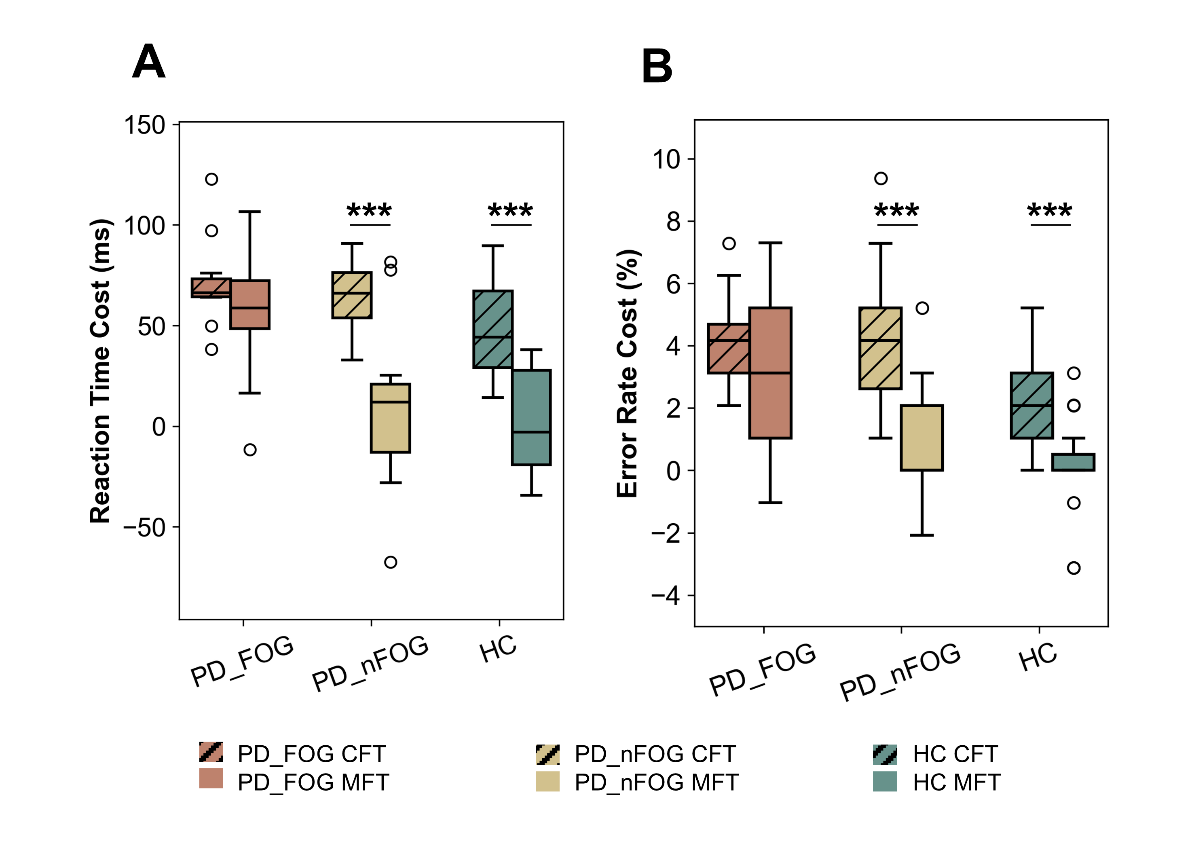
Figure S1 The results of the two-way repeated measures ANOVA for Flanker cost (RT and error rate) revealed significant main effects and interaction patterns.**

(A) For RT cost, there wa s a significant main effect of group (F(2,84)=16.66,*p*<0.001)and task type (F(1,84)=48.99,*p*<0.001), as well as a significant interaction between group and task type (F(2,84)=5.26,*p*=0.007).Post hoc analyses showed significant differences between CFT and MFT within both the PD-nFOG and HC groups (both *p*<0.001), but no significant difference within the PD-FOG group.

(B) For error rate cost, the main effects of group (F (2,84) = 11.77, *p*<0.001) and task type (F (1,84) = 30.30, *p*<0.001) were also significant. However, the interaction effect was not statistically significant (F (2,84) = 2.55, *p*=0.08). Post hoc comparisons indicated significant differences between CFT and MFT error rate costs for both PD-nFOG and HC groups (both *p*<0.01), while the PD-FOG group showed no significant differences across tasks.


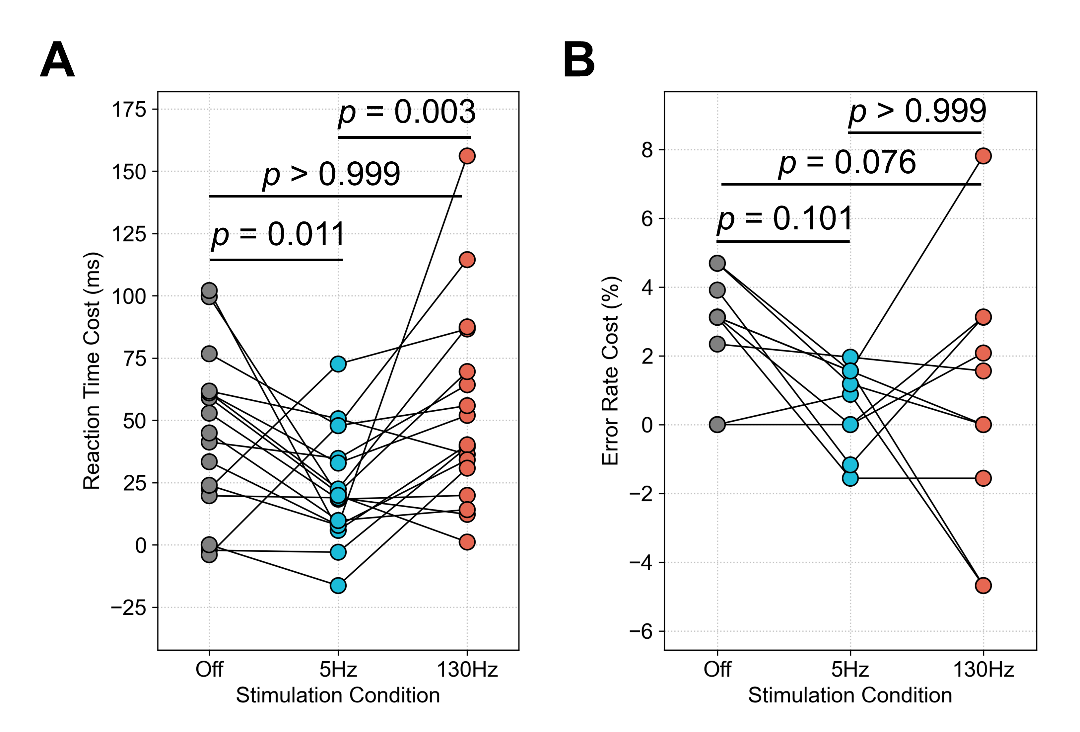


**Figure S2. Re-analysis of behavioral outcomes after removing statistical outliers.**

(A) Reaction time cost and (B) error rate cost across stimulation conditions (OFF, 5 Hz, and 130 Hz) after excluding statistical outliers using the interquartile range (IQR) method. Each line represents one subject.Theta-frequency stimulation significantly reduced RT cost compared to the OFF condition (*p* = 0.011), while 130 Hz stimulation showed no significant behavioral change (*p* > 0.999). For ER cost, no condition reached statistical significance after outlier removal.

**Supplementary Tables**

**Table S1. Demographic,** **clinical and neuropsychological characteristics of PD-FOG, PD-nFOG and HC groups in experiment 1.**

|  | **PD-FOG** | **PD-nFOG** | **HC** | ***p*-value** | ***p-*value**  **post-hoc analysis** | | |
| --- | --- | --- | --- | --- | --- | --- | --- |
| **Variables** | **(n=15)** | **(n=15)** | **(n=15)** | **ANOVA** | **PD-FOG vs PD-nFOG** | **PD-FOG vs HC** | **PD-nFOG vs HC** |
| **Demographic** |  |  |  |  |  |  |  |
| Age (years) | 63.73 ± 6.41 | 63.13 ± 6.64 | 63.93 ± 5.57 | 0.935 | >0.999 | >0.999 | >0.999 |
| Sex (M/F) ^a^ | 9/6 | 8/7 | 6/9 | 0.537 | 0.713 | 0.273 | 0.464 |
| Education (years) | 10.00 ± 3.57 | 10.07 ± 3.04 | 11.13 ± 2.80 | 0.547 | >0.999 | 0.990 | >0.999 |
| **Clinical** |  |  |  |  |  |  |  |
| Disease duration (years) | 9.60 ± 2.85 | 7.40 ± 2.72 | NA | NA | 0.039* | NA | NA |
| Side of onset (R/L/N) ^a^ | 8/6/1 | 7/8/0 | NA | NA | 0.715 | NA | NA |
| Hoehn-Yahr stage ^b^ | 3.03 ± 0.48 | 2.76 ± 0.53 | NA | NA | 0.136 | NA | NA |
| MDS-UPDRS-Ⅲ Med-ON ^b^ | 19.93 ± 9.32 | 15.33 ± 6.09 | NA | NA | 0.108 | NA | NA |
| MDS-UPDRS-Ⅲ Med-OFF | 46.40 ± 12.08 | 37.27 ±15.66 | NA | NA | 0.085 | NA | NA |
| FOG-Q ^b^ | 17.93 ± 3.85 | 2.67 ± 2.77 | NA | NA | < 0.001* | NA | NA |
| FOG-Q item 3 ^b^ | 3.13 ± 0.83 | 0 | NA | NA | < 0.001* | NA | NA |
| LEDD (mg) ^b^ | 986.28 ± 323.43 | 786.78 ± 600.51 | NA | NA | 0.134 | NA | NA |
| **Cognitive** |  |  |  |  |  |  |  |
| MMSE | 26.53 ± 1.46 | 27.80 ± 1.74 | 29.00 ± 1.25 | <0.001* | 0.094 | <0.001* | 0.230 |
| MoCA | 22.60 ± 1.55 | 23.53 ± 3.25 | 26.40 ± 2.64 | 0.002* | >0.999 | 0.002* | 0.025* |
| **Mood** |  |  |  |  |  |  |  |
| HAMA14 | 17.80 ± 5.61 | 13.87 ± 5.14 | NA | NA | 0.055 | NA | NA |
| HAMD17 | 18.53 ± 6.60 | 14.80 ± 4.59 | NA | NA | 0.083 | NA | NA |

Values are presented with means ± standard deviations or frequencies. For demographic variables, *p*-value refers to one-way ANOVA followed by post hoc Bonferroni's multiple comparisons tests for continuous variables and to ^a^ chi-squared test for categorical variables. Regarding clinical and mood variables, *p*-value refers to independent sample t test or ^b^ Mann-Whitney test for continuous variables, and to ^a^ chi-squared test for categorical variables. *Groups significantly different at *P* < 0.05.

ANOVA: analysis of variance, PD-FOG: Parkinson’s disease patients with freezing of gait, PD-nFOG: Parkinson’s disease patients without freezing of gait, HC: healthy control, MDS-UPDRS-Ⅲ Med-ON: 1 hour after taking the first dose of medications, MDS-UPDRS-Ⅲ Med-OFF: no dopaminergic taken for over 12 hours, FOG-Q: Freezing of Gait Questionnaire, LEDD: Levodopa equivalent daily dose, MMSE: Mini-Mental State Examination，MoCA: Montreal Cognitive Assessment, HAMA14: 14-item Hamilton Anxiety Rating Scale, HAMD24: 24-item Hamilton Depression Rating Scale, MMSE: mini-mental state examination.

**Table S2. Demographic, clinical and mood characteristics of PD-FOG, PD-nFOG and HC groups in experiment 2.**

|  | **PD-FOG** | **PD-nFOG** | **HC** | ***p*-value** | ***p*-value**  **post-hoc analysis** | | |
| --- | --- | --- | --- | --- | --- | --- | --- |
| **Variables** | **(n=22)** | **(n=20)** | **(n=22)** | **ANOVA** | **PD-FOG vs**  **PD-nFOG** | **PD-FOG vs HC** | **PD-nFOG vs HC** |
| **Demographic** |  |  |  |  |  |  |  |
| Age (years) | 62.00 ± 6.50 | 61.70 ± 5.52 | 63.09 ± 5.24 | 0.711 | >0.999 | >0.999 | >0.999 |
| Sex(M/F) ^a^ | 14/8 | 14/6 | 9/13 | 0.129 | 0.662 | 0.131 | 0.059 |
| Education (years) | 11.18 ± 3.58 | 10.78 ± 2.97 | 11.64 ± 2.72 | 0.671 | >0.999 | >0.999 | 0.799 |
| **Clinical** |  |  |  |  |  |  |  |
| Disease duration (years) | 9.18 ± 3.13 | 6.63 ± 2.10 | NA | NA | 0.004* | NA | NA |
| Side of Onset (R/L/N) ^a^ | 10/10/2 | 7/12/1 | NA | NA | 0.636 | NA | NA |
| Hoehn-Yahr stage ^b^ | 2.86 ± 0.41 | 2.80 ± 0.60 | NA | NA | 0.588 | NA | NA |
| MDS-UPDRS-Ⅲ Med-ON | 20.18 ± 8.60 | 16.30 ± 9.95 | NA | NA | 0.183 | NA | NA |
| MDS-UPDRS-Ⅲ Med-OFF | 43.32 ± 13.23 | 39.10 ±15.92 | NA | NA | 0.354 | NA | NA |
| FOG-Q ^b^ | 17.27 ± 4.15 | 1.15 ± 2.08 | NA | NA | <0.001* | NA | NA |
| FOG-Q item 3 ^b^ | 3.00 ± 0.98 | 0 | NA | NA | <0.001* | NA | NA |
| LEDD (mg) ^b^ | 984.80 ± 486.70 | 696.40 ± 544.20 | NA | NA | 0.023* | NA | NA |
| **Cognitive** |  |  |  |  |  |  |  |
| MMSE | 26.36 ± 1.50 | 27.75 ± 1.52 | 27.86 ± 1.58 | 0.006* | 0.032* | 0.010* | >0.999 |
| MoCA | 23.23 ± 2.11 | 25.05 ± 2.56 | 26.59 ± 1.71 | <0.001* | 0.023* | <0.001* | 0.070 |
| **Mood** |  |  |  |  |  |  |  |
| HAMA14 | 16.73 ± 6.97 | 12.75 ± 6.93 | NA | NA | 0.071 | NA | NA |
| HAMD24 | 17.91 ± 7.60 | 14.15 ± 6.82 | NA | NA | 0.100 | NA | NA |

Values are presented with means ± standard deviations or frequencies. For demographic variables, *p*-value refers to one-way ANOVA followed by post hoc Bonferroni's multiple comparisons tests or ^a^ chi-squared test. Regarding clinical and mood variables, *p*-value refers to independent sample t test, ^b^ Mann-Whitney test ^b^ or ^a^ chi-squared test. * Groups significantly different at *P* < 0.05.

ANOVA: analysis of variance, PD-FOG: Parkinson’s disease patients with freezing of gait, PD-nFOG: Parkinson’s disease patients without freezing of gait, HC: healthy control, MDS-UPDRS-Ⅲ Med-ON: 1 hour after taking the first dose of medications, MDS-UPDRS-Ⅲ Med-OFF: no dopaminergic taken for over 12 hours, FOG-Q: Freezing of Gait Questionnaire, LEDD: Levodopa equivalent daily dose, MMSE: Mini-Mental State Examination，MoCA: Montreal Cognitive Assessment, HAMA14: 14-item Hamilton Anxiety Rating Scale, HAMD24: 24-item Hamilton Depression Rating Scale.

**Table S3. Demographic and clinical characteristics of PD-FOG patients in experiment 3.**

| **Variables** | **PD-FOG (n = 18)** |
| --- | --- |
| **Demographic** |  |
| Age (years) | 63.22 ± 7.90 |
| Sex (M/F) | 9/11 |
| Education (years) | 11.94 ± 2.46 |
| **Clinical** |  |
| Disease duration (years) | 9.50 ± 4.83 |
| Side of Onset (R/L/N) | 7/10/1 |
| Hoehn-Yahr stage | 2.97 ± 0.58 |
| MDS-UPDRS-I | 11.33 ± 5.85 |
| MDS-UPDRS-II | 16.22 ± 8.67 |
| MDS-UPDRS-Ⅲ Stim-OFF/ Med-OFF | 45.33 ± 18.48 |
| FOG-Q | 16.17 ± 5.40 |
| FOG-Q item 3 | 3.06 ± 0.87 |
| LEDD (mg) | 476.22 ± 220.23 |
| **Optimal DBS parameter** |  |
| Left Amplitude (mA) | 2.43 ± 0.80 |
| Right Amplitude (mA) | 2.62 ± 0.76 |
| Left Pulse width (μs) | 73.33 ± 13.28 |
| Right Pulse width (μs) | 73.88 ± 12.43 |
| Frequency (Hz) | 124.17 ± 24.33 |
| **Cognitive** |  |
| MMSE | 26.39 ± 2.33 |
| MoCA | 23.94 ± 3.46 |
| **Mood** |  |
| HAMA14 | 14.39 ± 7.10 |
| HAMD24 | 10.78 ± 5.85 |

Values are presented with means ± standard deviations or frequencies. The UPDRS III assessment and the entire experiment were conducted OFF medication. The UPDRS III score was obtained during stimulation at the clinical frequency and without stimulation, administered in a randomized order across participants.

PD-FOG: Parkinson’s disease patients with freezing of gait, FOG-Q: Freezing of Gait Questionnaire, LEDD: Levodopa equivalent daily dose, MMSE: Mini-Mental State Examination，MoCA: Montreal Cognitive Assessment, HAMA14: 14-item Hamilton Anxiety Rating Scale, HAMD24: 24-item Hamilton Depression Rating Scale.

**Table S4. CFT and MFT behaviors of FOG, nFOG and HC groups in experiment 1.**

|  | **PD-FOG** | **PD-nFOG** | **HC** | ***p*-value** | ***p*-value**  **post-hoc analysis** | | |
| --- | --- | --- | --- | --- | --- | --- | --- |
| **Variables** | **(n=15)** | **(n=15)** | **(n=15)** | **ANOVA** | **PD-FOG vs**  **PD-nFOG** | **PD-FOG vs HC** | **PD-nFOG vs HC** |
| **CFT Reaction Time (ms)** |  |  |  |  |  |  |  |
| Congruent | 629.91 ± 115.40 | 591.52 ± 99.17 | 530.17 ± 55.70 | 0.019* | > 0.999 | 0.016* | 0.138 |
| Incongruent | 700.64 ± 121.15 | 655.72 ± 105.14 | 578.58 ± 63.27 | 0.006* | 0.862 | 0.005* | 0.064 |
| Reaction Time cost | 70.73 ± 19.20 | 64.20 ± 17.57 | 48.41 ± 22.46 | 0.011* | > 0.999 | 0.020* | 0.122 |
| **CFT Error Rates (%)** |  |  |  |  |  |  |  |
| Congruent | 3.41 ± 2.93 | 2.67 ± 1.88 | 3.41 ± 2.93 | < 0.001* | > 0.999 | < 0.001* | < 0.001* |
| Incongruent | 7.57 ± 2.80 | 6.88 ± 2.04 | 7.57 ± 2.80 | < 0.001* | > 0.999 | < 0.001* | < 0.001* |
| Error Rates cost | 4.17 ± 1.42 | 4.20 ± 2.32 | 4.17 ± 1.42 | 0.005* | > 0.999 | 0.004* | 0.032 |
| **MFT Reaction Time (ms)** |  |  |  |  |  |  |  |
| Congruent | 618.92 ± 111.19 | 563.63 ± 96.85 | 446.24 ± 62.82 | < 0.001* | 0.473 | < 0.001* | < 0.001* |
| Incongruent | 675.38 ± 125.41 | 571.00 ± 99.72 | 449.65 ± 69.30 | < 0.001* | 0.052 | < 0.001* | 0.002* |
| Reaction Time cost | 56.46 ± 29.12 | 7.38 ± 38.14 | 3.41 ± 25.35 | < 0.001* | 0.001* | < 0.001* | > 0.999 |
| **MFT Error Rates (%)** |  |  |  |  |  |  |  |
| Congruent | 2.91 ± 2.37 | 3.54 ± 2.11 | 0.90 ± 1.52 | 0.003* | > 0.999 | 0.029* | 0.002* |
| Incongruent | 5.90 ± 2.75 | 4.30 ± 3.07 | 0.97 ± 1.50 | < 0.001* | 0.433 | < 0.001* | < 0.001* |
| Error Rates cost | 2.99 ± 2.55 | 0.76 ± 1.87 | 0.07 ± 1.69 | 0.001* | 0.032* | 0.002* | 0.886 |

Values are presented with means ± standard deviations. Statistical significance was assessed using a repeated-measures ANOVA followed by Bonferroni's post hoc comparisons (adjusted p-values shown). PD-FOG: Parkinson’s disease patients with freezing of gait, PD-nFOG: Parkinson’s disease patients without freezing of gait, HC: healthy control, Reaction Time cost cost: mean Reaction Time of incongruent minus mean Reaction Time of congruent, Error Rates cost: mean Error Rates of incongruent minus mean Error Rates of congruent

**Table S5. MFT behaviors of PD-FOG, PD-nFOG and HC groups in experiment 2.**

|  | **PD-FOG** | **PD-nFOG** | **HC** | ***p*-value** | ***p*-value**  **post-hoc analysis** | | |
| --- | --- | --- | --- | --- | --- | --- | --- |
| **Variables** | **(n=22)** | **(n=20)** | **(n=22)** | **ANOVA** | **PD-FOG vs**  **PD-nFOG** | **PD-FOG vs HC** | **PD-nFOG vs HC** |
| **MFT Reaction Time (ms)** |  |  |  |  |  |  |  |
| Congruent | 590.31 ± 140.09 | 539.67 ± 94.54 | 488.29 ± 95.04 | 0.014* | 0.319 | 0.012* | 0.308 |
| Incongruent | 640.38 ± 164.07 | 545.34 ± 103.79 | 491.04 ± 97.23 | < 0.001* | 0.046* | < 0.001* | 0.350 |
| Reaction Time cost | 50.07 ± 36.64 | 5.66 ± 32.46 | 2.76 ± 20.60 | < 0.001* | < 0.001* | < 0.001* | 0.949 |
| **MFT Error Rates (%)** |  |  |  |  |  |  |  |
| Congruent | 2.89 ± 2.73 | 1.93 ± 2.69 | 0.95 ± 1.70 | 0.034* | 0.405 | 0.026* | 0.0391 |
| Incongruent | 5.21 ± 3.37 | 2.86 ± 2.98 | 1.56 ± 1.84 | < 0.001* | 0.024* | < 0.001* | 0.296 |
| Error Rates cost | 2.32 ± 2.36 | 0.94 ± 2.27 | 0.61 ± 1.87 | 0.028* | 0.106 | 0.031* | 0.881 |

Values are presented with means ± standard deviations. Statistical significance was assessed using a repeated-measures ANOVA followed by Bonferroni's post hoc comparisons (adjusted p-values shown). PD-FOG: Parkinson’s disease patients with freezing of gait, PD-nFOG: Parkinson’s disease patients without freezing of gait, HC: healthy control, Reaction Time cost: mean Reaction Time of incongruent minus mean Reaction Time of congruent, Error Rates cost: mean Error Rates of incongruent minus mean Error Rates of congruent.

**Table S6. Clusters of activation for the conflict effect (incongruet > congruent) during MFT in experiment 2.**

| **Cluster** | **Cluster size ^a^** | **Hemisphere** | **Region** | **Brodmann**  **area** | **Peak T/F value** | **MNI coordinates** | | |
| --- | --- | --- | --- | --- | --- | --- | --- | --- |
|  |  |  |  |  |  | **X** | **Y** | **Z** |
| **PD-FOG** | | | | | | | | |
| 1 | 2258 | L/R | Presupplementary motor area, Anterior cingulate cortex, Medial superior frontal gyrus | 9/32 | 6.71 | 4 | 32 | 34 |
| 2 | 314 | R | superior frontal gyrus, Middle frontal gyrus | 6 | 6.31 | 46 | 38 | 28 |
| 3 | 499 | R | Insular, Inferior frontal gyrus | 13/47 | 7.12 | 44 | 24 | -10 |
| 4 | 473 | L | Inferior occipital gyrus, Fusiform gyrus | 18/37 | 6.95 | -46 | -66 | -14 |
| 5 | 225 | L | Middle temporal gyrus | 21 | 6.91 | -54 | -28 | 0 |
| 6 | 91 | L/R | Medial orbitofrontal cortex | 32 | 6.71 | -4 | 42 | -14 |
| 7 | 355 | L | Insular, Inferior frontal gyrus | 13/47 | 6.63 | -50 | 32 | -6 |
| 8 | 1372 | R | Middle/inferior temporal gyrus,  Middle/inferior occipital gyrus | 21/37 | 6.41 | 56 | -52 | -16 |
| 9 | 259 | L/R | Posterior cingulate cortex | 31 | 6.37 | 4 | -36 | 36 |
| 10 | 415 | L | Middle frontal gyrus | 9 | 5.93 | -32 | 12 | 34 |
| 11 | 280 | R | Middle occipital gyrus,  Angular gyrus | 19/39 | 5.81 | 36 | -72 | 30 |
| 12 | 321 | L | Inferior parietal lobe | 40 | 5.47 | -30 | -52 | 40 |
| 13 | 101 | R | Inferior frontal gyrus, triangular part | 45 | 5.20 | 58 | 24 | 10 |
| 14 | 283 | L | Middle occipital gyrus | 19 | 5.20 | -30 | -92 | 20 |
| 15 | 220 | L | Superior parietal lobe | 7 | 4.67 | -24 | -72 | 40 |
| 16 | 167 | R | Inferior parietal lobe,  Supramarginal gyrus | 40 | 4.58 | 58 | -44 | 38 |
| **PD-nFOG** | | | | | | | | |
| 1 | 164 | L | Superior occipital gyrus,  Middle occipital gyrus,  Superior parietal lobe | 19 | 6.25 | -22 | -68 | 44 |
| 2 | 80 | L | Middle occipital gyrus | 18/19 | 4.78 | -36 | -94 | 2 |
| **HC** | | | | | | | | |
| 1 | 364 | L | Inferior temporal gyrus,  Inferior occipital gyrus,  Fusiform Gyrus | 37/19 | 6.01 | -48 | -60 | -14 |
| 2 | 146 | R | Inferior temporal gyrus,  Fusiform Gyrus | 19 | 5.72 | 56 | -62 | -14 |
| 3 | 189 | L | Middle occipital gyrus | 19 | 5.46 | -28 | -78 | 20 |
| 4 | 82 | R | Middle occipital gyrus | 19 | 5.34 | 30 | -76 | 32 |
| 5 | 91 | L | Superior parietal lobe | 7 | 4.54 | -30 | -58 | 52 |
| **Activation differences across the three groups** | | | | | | | | |
| 1 | 1061 | L/R | Medial superior frontal gyrus  Anterior cingulate cortex,  Presupplementary motor area | 9/32 | 27.97 | 0 | 40 | 24 |
| 2 | 71 | L/R | Posterior cingulate gyrus | 24 | 16.08 | 2 | -22 | 42 |
| 3 | 539 | R | Middle temporal gyrus,  Superior temporal gyrus,  Inferior temporal gyrus, | 21 | 15.83 | 64 | -8 | -16 |
| 4 | 221 | R | Middle Frontal Gyrus | 9 | 15.64 | 38 | 24 | 36 |
| 5 | 111 | L | Middle temporal gyrus | 21 | 14.57 | -54 | -30 | -8 |

^a^ Number of voxels exceeding a voxel-level threshold of p < 0.001, with clusters meeting a cluster-size threshold of p < 0.05 (FWE-corrected).

MNI: Montreal Neurological Institute standard space.

**Table S7. Frequency specific effects of STN-DBS on conflict resolution and motor symptom.**

|  | **Stimulation frequency** | | | ***p*-value** | ***p*-value**  **post-hoc analysis** | | |
| --- | --- | --- | --- | --- | --- | --- | --- |
| **Variables** | **OFF** | **Theta** | **High gamma** | **ANOVA** | **Stim-OFF vs**  **Theta** | **Stim-OFF vs**  **High gamma** | **Theta vs**  **High gamma** |
| **Reaction Time (ms)** |  |  |  |  |  |  |  |
| Congruent | 703.57 ± 145.32 | 712.36 ± 153.96 | 696.39 ± 121.84 | 0.513 | 0.952 | 0.952 | > 0.999 |
| Incongruent | 752.11 ± 160.47 | 736.04 ± 162.49 | 758.85 ± 138.24 | 0.066 | 0.730 | 0.730 | 0.059 |
| Reaction Time cost | 48.54 ± 36.03 | 23.68 ± 21.57 | 62.47 ± 53.44 | < 0.001* | 0.008* | > 0.999 | 0.001* |
| ^†^Reaction Time cost | 44.33 ± 32.27 | 23.46 ± 22.22 | 53.80 ± 39.98 | 0.002* | 0.011* | > 0.999 | 0.003* |
| **Error Rates (%)** |  |  |  |  |  |  |  |
| Congruent | 6.58 ± 11.05 | 10.01 ± 16.29 | 10.74 ± 13.87 | 0.203 | > 0.999 | 0.470 | 0.547 |
| Incongruent | 12.67 ± 18.08 | 11.62 ± 16.28 | 15.58 ± 19.50 | 0.161 | 0.240 | > 0.999 | 0.634 |
| Error Rates cost | 6.10 ± 7.73 | 1.61 ± 2.89 | 4.83 ± 9.51 | 0.071 | 0.112 | 0.287 | > 0.999 |
| ^†^Error Rates cost | 2.81 ± 1.66 | 0.60 ±1.23 | 0.68 ± 3.79 | 0.030* | 0.101 | 0.076 | > 0.999 |
| **Motor symptom** |  |  |  |  |  |  |  |
| MDS-UPDRS-Ⅲ  Med-OFF | 45.33 ± 18.48 | 42.94 ±16.97 | 21.50 ±9.09 | < 0.001* | 0.287 | < 0.001* | < 0.001* |

Values are presented as mean ± standard deviation. Statistical significance was assessed using a repeated-measures Friedman test followed by Dunn’s post hoc comparisons (adjusted p-values shown). ^†^ Indicates results re-analyzed after excluding statistical outliers identified using the IQR method (one subject in the 130 Hz condition for RT cost; 8 subjects across conditions for error rate cost). Reaction Time cost: mean Reaction Time of incongruent minus mean Reaction Time of congruent, Error Rates cost: mean Error Rates of incongruent minus mean Error Rates of congruent. MDS-UPDRS-Ⅲ Med-OFF: no dopaminergic taken for over 12 hours.

**Table S8.** **Cluster activation patterns of whole-brain response in PD-FOG patients under STN-DBS at different frequencies in experiment 3.**

| **Cluster** | **Cluster size ^a^** | **Hemisphere** | **Region** | **Brodmann**  **area** | **Peak T/F value** | **MNI coordinates** | | | |  |  |  |
| --- | --- | --- | --- | --- | --- | --- | --- | --- | --- | --- | --- | --- |
|  |  |  |  |  |  | **X** | **Y** | **X** | |  |  |  |
| **Theta stimulation > OFF** | | | | | | | | | | | |  |
| 1 | 158 | L/R | Medial superior frontal gyrus | 6/8/9/10 | 13.63 | 6 | 48 | 42 | |  |  |  |
| 2 | 282 | R | Middle frontal gyrus,  Presupplementary motor area,  Anterior cingulate cortex, | 6 | 10.18 | 21 | 3 | 60 | |  |  |  |
| 3 | 154 | L | Angular gyrus | 39 | 10.15 | -39 | -60 | 33 | |  |  |  |
| 4 | 121 | L/R | Posterior cingulate gyrus | 31 | 9.37 | 0 | -36 | 39 | |  |  |  |
| 5 | 80 | L | Middle frontal gyrus | 6 | 8.29 | -30 | 9 | 54 | |  |  |  |
| 6 | 192 | R | Angular gyrus | 39 | 7.95 | 42 | -75 | 36 | |  |  |  |
| 7 | 372 | R | Cuneus | 19 | 7.52 | 9 | -63 | 9 | |  |  |  |
| 8 | 57 | L | Inferior frontal gyrus, triangular part | 45/9 | 7.33 | -48 | 12 | 33 | |  |  |  |
| 9 | 99 | L | Middle frontal gyrus | 10 | 7.26 | 42 | 57 | -6 | |  |  |  |
| 10 | 54 | R | Posterior lobe of cerebellum | - | 6.62 | 21 | -69 | -33 | |  |  |  |
| 11 | 85 | L | Orbitofrontal cortex | 10 | 6.48 | -6 | 51 | -3 | |  |  |  |
| 12 | 74 | L | Posterior lobe of cerebellum | - | 6.06 | -39 | -75 | -33 | |  |  |  |
| 13 | 83 | L | Inferior frontal gyrus, triangular part | 45/11 | 4.16 | -45 | -48 | -6 | |  |  |  |
| **High gamma stimulation > OFF** | | | | | | | | | | |  |  |
| 1 | 23 | - | Vermis of cerebellum | - | 8.59 | 12 | -66 | -36 | |  |  |  |
| 2 | 40 | L | Globus pallidus internus | - | 6.62 | -21 | -6 | -3 | |  |  |  |
| 3 | 21 | L | Anterior lobe of cerebellum | - | 6.24 | -12 | -48 | -39 | |  |  |  |
| 4 | 76 | R | Thalamus | - | 6.60 | 15 | -18 | 18 | |  |  |  |
| 5 | 72 | L | Thalamus | - | 6.51 | -9 | -18 | 12 | |  |  |  |
| 6 | 41 | L | Insular | 13 | 4.64 | -36 | -3 | 15 | |  |  |  |
| **High gamma stimulation < OFF** | | | | | | | | |  |  |  |  |
| 1 | 786 | L/R | Medial superior frontal gyrus，  Presupplementary motor area,  Anterior cingulate cortex, | 6/8/9/10 | 9.21 | -9 | 42 | 33 | |  |  |  |
| 2 | 273 | R | Inferior frontal gyrus, triangular part | 45/9 | 7.32 | 51 | 22 | 16 | |  |  |  |
| 3 | 256 | R | Precentral gyrus, Middle frontal gyrus | 4 | 8.19 | 50 | 6 | 34 | |  |  |  |
| 4 | 449 | L | Inferior frontal gyrus, triangular part | 45 | 6.75 | -48 | 27 | 0 | |  |  |  |
| 5 | 198 | L | Middle temporal gyrus | 21 | 5.94 | -36 | 21 | -39 | |  |  |  |
| 6 | 201 | R | Middle temporal gyrus | 21 | 4.24 | 60 | -30 | -6 | |  |  |  |
| **Theta stimulation > High gamma stimulation** | | | | | | | | | |  |  |  |
| 1 | 1219 | L/R | Medial superior frontal gyrus  Presupplementary motor area,  Anterior cingulate cortex, | 6/8/9/10 | 12.31 | -3 | 45 | 48 | |  |  |  |
| 2 | 546 | L | Inferior frontal gyrus, triangular part | 45 | 10.40 | -39 | 18 | 24 | |  |  |  |
| 3 | 492 | L | Precentral gyrus，Middle frontal gyrus，Postcentral gyrus | 6/4/8 | 10.01 | -18 | -15 | 72 | |  |  |  |
| 4 | 966 | R | Superior parietal lobe, Angular gyrus | 38/39/40 | 11.33 | 54 | -6 | -36 | |  |  |  |
| 5 | 645 | L/R | Cuneus, calcarine sulcus | 19 | 8.01 | -5 | -75 | 12 | |  |  |  |
| 6 | 65 | R | Posterior cingulate gyrus，precuneus | 31 | 6.86 | 6 | -45 | 39 | |  |  |  |
| 7 | 46 | L | Middle temporal gyrus | 21 | 4.55 | -54 | -12 | -15 | |  |  |  |
| 8 | 107 | L | Angular gyrus | 39 | 4.38 | -36 | -69 | 33 | |  |  |  |
| **Theta stimulation > High gamma stimulation** | | | | | | | | |  | | | |
| 1 | 30 | L | Globus pallidus internus | - | 6.67 | 30 | -21 | -6 | |  |  |  |
| 2 | 47 | R | Globus pallidus internus | - | 6.64 | 21 | -3 | 3 | |  |  |  |
| 3 | 53 | R | Thalamus | - | 4.46 | 6 | -18 | 15 | |  |  |  |
| 4 | 32 | L | Thalamus | - | 3.96 | -4 | -22 | 10 | |  |  |  |

^a^ Number of voxels exceeding a voxel-level threshold of p < 0.001, with clusters meeting a cluster-size threshold of p < 0.05 (FWE-corrected).

MNI: Montreal Neurological Institute standard space.
